# An Easily Overlooked Presentation of Malignant Psoas Abscess: Hip Pain

**DOI:** 10.1155/2015/410872

**Published:** 2015-01-22

**Authors:** Ayhan Askin, Korhan Baris Bayram, Umit Secil Demirdal, Merve Bergin Korkmaz, Alev Demirbilek Gurgan, Mehmet Fatih Inci

**Affiliations:** ^1^Katip Celebi University, Ataturk Training and Research Hospital, Physical Medicine and Rehabilitation Clinic, 35360 Izmir, Turkey; ^2^Katip Celebi University, Ataturk Training and Research Hospital, Radiology Clinic, 35360 Izmir, Turkey

## Abstract

Psoas abscess is a rare infectious disease with nonspecific clinical presentation that frequently causes a diagnostic difficulty. Its insidious onset and occult characteristics can cause diagnostic delays. It is classified as primary or secondary. Staphylococcus aureus is the most commonly causative pathogen in primary psoas abscess. Secondary psoas abscess usually occurs as a result of underlying diseases. A high index of clinical suspicion, the past and recent history of the patient, and imaging studies can be helpful in diagnosing the disease. The delay of the treatment is related with high morbidity and mortality rates. In this paper, 54-year-old patient with severe hip pain having an abscess in the psoas muscle due to metastatic cervical carcinoma is presented.

## 1. Introduction

Psoas abscess (PA) is a rarely observed infective clinical condition, which is difficult to diagnose and therefore may cause morbidity and mortality [1]. Psoas muscle is located in retroperitoneal space and extends from lateral borders of 12th thoracic vertebra and all lumbar vertebra to femur trochanter minor. It is closely adjacent to organs such as kidneys, sigmoid colon, jejunum, appendix, pancreas, abdominal aorta, and ureter [2]. Due to anatomic localization and significant adjacency of the muscle, PA may demonstrate a variable clinical symptomatology and may have an insidious course, and there may be treatment challenges when diagnosed [2, 3].

The classical clinical presentation of the disease is fever, back pain, and walking abnormality (limping) [3]. There are numerous reports related to PA in the literature [4–6]. However, patients applying with subacute hip pain and walking abnormality are rather rarely observed by physicians engaged in musculoskeletal system. Herein a patient who had abscess which developed in psoas muscle secondary to multiple metastasis of cervical carcinoma and who applied to our outpatient clinic with complaint of hip pain was presented.

## 2. Case Report

A 54-year-old female patient was admitted to our outpatient clinic with complaints of left hip pain markedly increasing for 10 days and difficulty in walking. She stated that she had hip pain for about 2 months, and she was treated by many physicians. Patient had difficulty in load transfer during walking and therefore had ambulation difficulty. She defined loss of appetite and sometimes fever. She did not define low back pain, radicular symptoms, neuropathic complaints, trauma, rash, aphtha, diarrhea, arthritis, abdominal pain, a recent infection, a history of intramuscular injection, weight loss, history of eating fresh cheese, and history of tuberculosis. Patient received chemotherapy and radiotherapy 3 years ago due to cervical cancer, and she was a diabetes patient using insulin.

In patient's evaluation, her arterial blood pressure measurement was 110/70 mmHg, her fever was 37.1°C, and her pulse rate was 95/minute. Patient appeared to be pale and tired, and she was mobilized on wheelchair. Due to pain, she could not bear load on left lower extremity, she needed assistance in walking, and she had difficulty in transfer activities. Left hip joint was flexed to 50 degrees. Passive joint space could not be evaluated. She defined tenderness at trochanter major by palpation. Right hip and vertebral movements were extended and painless. Spasm was detected in left paravertebral muscles at lumbar region. No neurological deficit was detected, and no pathological reflex was present. She had no edema in lower extremities, and all pulses could be measured.

**Figure 1:**
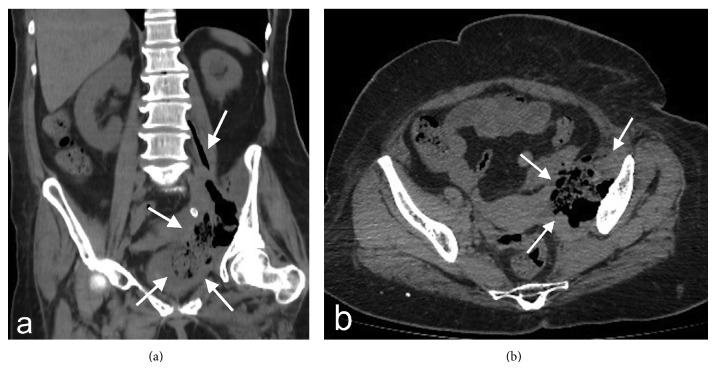
In coronal reformat (a) and axial (b) section computerized tomography examination, appearance is observed in concordance with abscess, extending from left iliopsoas level to inferior pelvic space and containing air and intense fluid densities (white arrows).

After initial evaluation, lumbar, pelvis, and left hip roentgenogram and ultrasonography (US) for hip joint and gluteal region were planned. Roentgenograms were normal except mild osteodegenerative findings at lumbar vertebra. Slight amount of fluid was observed at left trochanteric bursa in US. Hip magnetic resonance examination (MR) was requested for clear evaluation of hip joint and adjacent structures. However, it could not be performed since the patient could not be positioned due to pain. In her laboratory examination, the following was detected: leucocytes: 17.86 (4–10) k/uL, hemoglobin: 7.3 (11–16) g/dL, sedimentation (ESR): 110 mm/hour, creatinine: 1.56 (0.6–1.1) mg/dL, C-reactive protein (CRP): 31.18 (0.01–0.82) mg/dL, and brucella agglutination: negative. In her urinalysis, protein (+++) and leucocytes 1500 p/HPF were found. Patient had history of radiotherapy and chemotherapy due to cervical cancer, and urgent nonopaque abdominal computerized tomography (CT) was performed for the patient with current findings and prediagnosis of metastasis and abscess. Soft tissue lesion was observed consistent with abscess at left iliac fossa in iliopsoas muscle, filling paravertebral region with lucent gas inside (Figures 1(a) and 1(b)). PA was detected and she was hospitalized in urology clinic for treatment. Blood culture was negative and, following preferred metronidazole and piperacillin/tazobactam treatment, patient had an operation. PA, ureter, and sigmoid colon perforation were detected. In pathological examination of nephroureterectomy and sigmoid colon resection material, ureter and sigmoid colon metastasis of squamous cell carcinoma were detected.

## 3. Discussion 

Our report is a case, which can be rarely observed due to patient's presenting with subacute hip pain and being diagnosed with PA secondary to cervical metastasis. PA-related reports in literature are usually case reports or case series. The incidence was reported in 1992 as 12/100000, and yet no current data is available. Increased incidence is expected due to increased disease awareness, development of diagnostic approaches and devices, increased number of multisystemic diseases, and malignancies [4, 5].

The disease is classified as primary or secondary. Primary PA composes 30% of all cases and develops generally via diffusion of bacteria from an insidious focus by hematogenous or lymphatic routes. Secondary cases emerge as the result of local diffusion from adjacent infected tissues [2]. The prevalence of primary PA was low in developed countries; however it increases due to increased number of immunocompromised patients. The most frequently responsible microorganisms are reported as* Staphylococcus aureus*,* Escherichia coli*,* Bacteroides* species, and* Mycobacterium tuberculosis* [6]. The most frequently observed diseases associated with secondary PA are Crohn's disease, appendicitis, ulcerative colitis, diverticulitis, colorectal carcinomas, urinary system infection and instrumentation, vertebral infections and osteomyelitis, and septic arthritis [6, 7]. In their retrospective case series, Wong et al. [8] detected secondary PA in 23 of 42 patients and reported the most frequently observed secondary cause as infective spondylitis and spondylodiscitis. In one patient, they detected a case of infection secondary to cervix carcinoma. Dietrich et al. [1] detected secondary PA in 80% of their case series and reported the most frequent cause as spondylodiscitis. Kim et al. [7] detected secondary PA in 61% of a series of 105 patients and the most frequent cause as spondylodiscitis. In the literature, many cases are reported as cervix cancer metastasis to psoas muscle [9, 10]. Our case is rare due to presentation of cervix cancer metastasis with psoas abscess and development of PA secondary to colon and ureter metastasis of cervix cancer.

The classical triad of the disease, fever, back pain, and limping are not observed in each case [8]. Dietrich et al. [1] detected the clinical triad in 5% of their patients, and Lee et al. [11] detected it in 9% of their patients. As patients may initially present nonspecific symptomatology as malaise, fatigue, and subfebrile fever, they may demonstrate a more severe presentation such as abdominal-groin pain, low back pain, hip pain, difficulty in hip movements, high fever, loss of appetite, and weight loss. Complaints about low back-hip region are frequently observed due to extension of psoas muscle and pain from L2-3-4 roots [2]. Therefore, cases primarily presenting musculoskeletal complaints may apply to orthopedics and rehabilitation medicine outpatient clinics. In a retrospective study, it was detected that almost half of 37 patients included in the study had low back-hip pain and that 13 patients applied to orthopedics outpatient clinic directly [8]. Diagnosis of patients is delayed due to this uncertain clinical symptomatology. Hamano et al. [12] reported that prediagnosis symptom duration of patients could vary from 1 day to 63 days, and Wong et al. reported that it could vary from 1 day to 3 months. Our patient had complaints for about 2 months, and she was evaluated several times by orthopedics, sports medicine due to initially mild hip pain, and she received medical treatment when she applied to us.

In diagnosis, laboratory and imaging methods are also used besides clinical evaluation. For infectious process, complete blood count, ESR, CRP, and complete urinalysis should be initially requested in laboratory examination [2, 3]. Leukocytosis, CRP and ESR elevation, anemia, and growth in blood culture may be detected. Direct abdominal graphy on standing position and pelvis, lumbar vertabrae, and lung roentgenograms may be beneficial according to clinical histories of patients. US, CT, and MR imaging are the most valuable imaging methods in diagnosis [3, 13]. Although US is a partially cheaper examination, which has no radiation effect and which is convenient to administer, it is operator dependent. Moreover, positive findings may be obtained only in 60% of the cases due to difficult demonstration of retroperitoneal space and intensity of flatus [3]. It was demonstrated that MR is more sensitive than CT in diagnosis of intra-abdominal abscesses. CT may provide false negative results in diagnosis of especially nonair containing abscesses [2]. In our patient, MR examination was considered primarily in diagnosis since it had additional diagnosis value also in musculoskeletal pathologies. However, BT was performed due to positioning problem, and abscess findings were demonstrated in psoas muscle.

Infections (septic arthritis of hip, necrotizing fasciitis of psoas muscle, pyelonephritis, pelvic inflammatory disease, appendicitis, osteomyelitis, and epidural abscesses), vascular pathologies (femur avascular necrosis, aneurysms), retroperitoneal malignancies, inflammatory bowel diseases, urolithiasis, and discopathies should be suggestive in differential diagnosis of the disease [14]. Since most diseases included in differential diagnosis compose especially musculoskeletal complaints, they should be carefully evaluated, and this diagnosis should be certainly kept in mind in laboratory tests to be requested and in imaging methods. As in our case, if not evaluated in detail, patients may be initially evaluated as a primary skeletal system patient. Since delayed diagnosis may cause increased morbidity and mortality, time should not be lost with unnecessary examinations.

In treatment, appropriate antibiotherapy, percutaneous or open drainage, and the treatment of a secondary cause, if detected, should be the basic approach [3, 15]. Mortality is low with early diagnosis and appropriate treatment. Mortality rate varies from 5% to 11% [2, 7].

In conclusion, since it may have a nonspecific clinical onset, it is important firstly to suspect the disease. Possible secondary causes should certainly be taken into account. A well-performed physical examination is important to detect causes for local or referred pain. When it is considered that mortality rates decrease by early diagnosis of the disease, it is very important for physicians involved in musculoskeletal system to keep PA pre-diagnosis in mind.
